# Induction of the STING-dependent DNA damage pathway by cucurbitacin B enhances immunotherapy efficacy in osteosarcoma

**DOI:** 10.22038/ijbms.2025.86376.18663

**Published:** 2025

**Authors:** Bin Luo, Qing Lu, Qiang Wang, Yingchao Shen, Yiming Miao, Yong Ma

**Affiliations:** 1 Department of Orthopaedics, Changshu Hospital Affiliated to Nanjing University of Chinese Medicine, Changshu 215500, China; 2 Department of Orthopaedics, The Third People’s Hospital of Xiangcheng District, Suzhou 215100, China; 3 Institute of Traumatology & Orthopedics and Laboratory of New Techniques of Restoration & Reconstruction of Orthopedics and Traumatology,; 4 Nanjing University of Chinese Medicine, Nanjing, 210023, China; 5 Department of Traumatology & Orthopedics, Affiliated Hospital of Nanjing University of Chinese Medicine, Nanjing, 210004, China

**Keywords:** cGAS-STING pathway, Cucurbitacin B, DNA damage, Osteosarcoma, PD-L1

## Abstract

**Objective(s):**

Osteosarcoma (OS) is a highly aggressive bone tumor with limited therapeutic options. Cucurbitacin B (CuB), a natural compound derived from Cucurbitaceae plants, has demonstrated antitumor activity in various malignancies; however, its mechanisms in OS remain unclear. This study aims to elucidate the antitumor effects of CuB in OS and explore its molecular mechanisms.

**Materials and Methods:**

MG63 and K7M2 OS cells were treated with CuB, and cell viability was assessed using the cell counting kit-8 (CCK8) assay. Colony formation assays were employed to evaluate proliferation, while flow cytometry was used to analyze apoptosis and cell cycle distribution. DNA damage was determined by immunofluorescence staining and comet assay. Western blotting was used to detect proteins involved in activating the stimulator of the interferon genes (STING) pathway. *In vivo *OS xenograft models were established to monitor tumor growth and immune responses, and the therapeutic efficacy of combination treatment with anti-programmed death-ligand 1 (PD-L1) was evaluated.

**Results:**

CuB inhibited OS cell proliferation, induced apoptosis, and caused G2/M cell cycle arrest. It activated the STING pathway and induced DNA damage. *In vivo*, CuB reduced tumor growth and metastasis, enhanced CD8+ and CD4+ T cell infiltration, and reduced regulatory T cells (Tregs) and myeloid-derived suppressor cells (MDSCs). Combination with anti-PD-L1 further suppressed tumor growth.

**Conclusion:**

CuB exerts anti-OS effects by inducing DNA damage, activating the STING pathway, enhancing immune responses, and synergizing with anti-PD-L1, highlighting its therapeutic potential.

## Introduction

Osteosarcoma (OS) is a rare but highly aggressive bone malignancy that primarily affects pediatric and adolescent populations (1). Despite advancements in multimodal therapies, including surgery, chemotherapy, and radiation, the prognosis for metastatic OS remains dismal, with a 5-year survival rate below 30% (2). OS is characterized by its high local invasiveness and strong metastatic potential, often presenting symptoms such as localized pain, palpable masses, and pathological fractures (3, 4). Current treatment strategies, though standard, remain insufficient to significantly improve long-term survival outcomes, highlighting the urgent need for novel therapeutic approaches (5).

Natural products have emerged as promising anticancer agents due to their diverse pharmacological properties and favorable safety profiles (6). Cucurbitacin B (CuB), a tetracyclic triterpenoid derived from Cucurbitaceae plants, has demonstrated potent antitumor effects across multiple malignancies, including lung, liver, and breast cancers, by modulating inflammation, oxidative stress, cell cycle progression, and apoptosis (7-10). Recent investigations propose that CuB inhibits OS progression by regulating M2 macrophage polarization via the PI3K/AKT signaling pathway (11). Moreover, CuB suppresses OS cell proliferation and induces apoptosis through the JAK2/STAT3 and MAPK pathways (12). CuB also works synergistically with methotrexate in low doses and induces DNA damage in various cancer cells (13). Interestingly, at low doses, CuB shows minimal toxicity to normal cells and animals while selectively exerting cytotoxic effects on cancer cells, further supporting its therapeutic potential (8). However, the mechanisms of CuB-induced DNA damage in OS and its potential impact on tumor immunity remain unexplored.

DNA damage and defective DNA repair mechanisms are intricately linked to tumor initiation and progression (14, 15). Targeting DNA damage response pathways has proven effective in sensitizing OS to conventional therapies, such as doxorubicin, which exerts its antitumor effects through DNA damage induction (16). Notably, the cyclic GMP-AMP synthase (cGAS)/stimulator of interferon genes (STING) pathway, a key intracellular DNA sensor, links DNA damage to immune activation by detecting cytosolic DNA fragments and triggering type I interferon release, which promotes immune cell recruitment and T cell activation(17, 18). Immunotherapy has revolutionized cancer treatment, offering durable responses to various malignancies (19). However, tumor cells evade immunity by up-regulating PD-L1, which binds to PD-1 on T cells, causing exhaustion and immune escape (20, 21). Anti-PD-L1 therapy shows promise in OS, with ongoing research assessing its efficacy alone or in combination with other treatments (22). Given the expanding applications of immunotherapy, its potential role in OS treatment warrants further exploration.

This study demonstrates CuB’s ability to inhibit OS cell proliferation, induce apoptosis, arrest the cell cycle, and trigger DNA damage, leading to cGAS/STING pathway activation *in vitro*. *In vivo*, CuB suppresses OS xenograft growth, enhances DNA damage, and activates STING signaling in tumors. Additionally, CuB promotes immune cell infiltration and strengthens anti-PD-L1 therapy. These findings underscore CuB’s potential as a treatment for OS, enhancing antitumor immunity by inducing DNA damage and triggering the STING pathway.

## Materials and Methods

### Reagents

**Figure 1:**
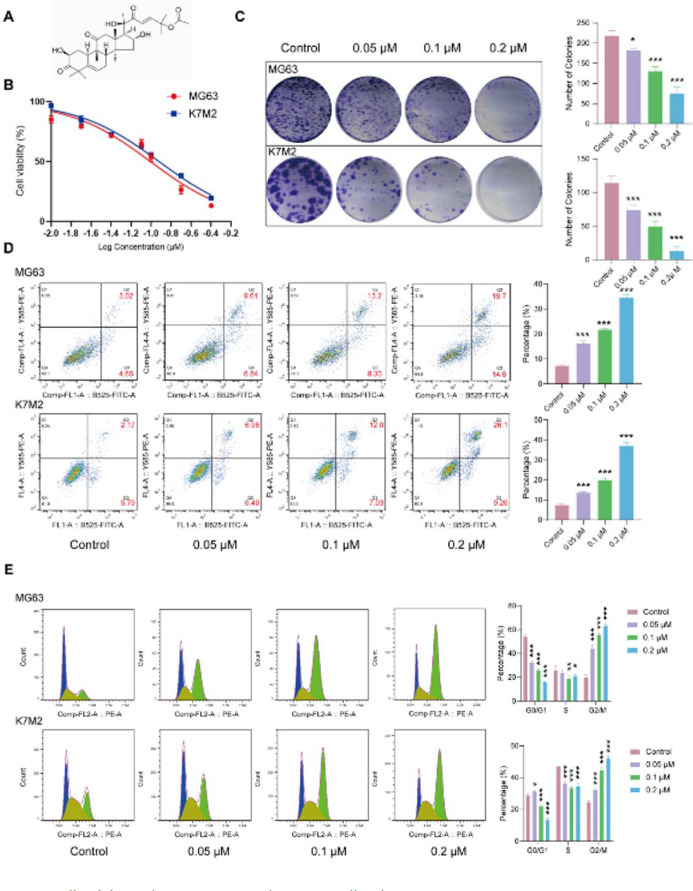
CuB suppresses OS cell viability, induces apoptosis, and promotes cell cycle arrest

Cucurbitacin B (C_32_H_46_O_8_, CAS number: 6199-67-3, HY-N0416, Purity > 99.91%; Figure 1A) was obtained from MedChemExpress (USA). Antibodies against gamma-H2A histone family member X (γ-H2AX) (#7631), cGAS (#79978), phospho (p)- TANK-binding kinase-1 (TBK1) (#5483), TBK1 (#3504), p-STING (#72971), STING (#13647), p-interferon regulatory factor (IRF3) (#4947), interferon regulatory factor (IRF3) (#4302), Ki67 (#9129), and GAPDH (#2118) were obtained from Cell Signaling Technology (Boston, USA).

### Cells

The human OS cell line MG63 and the murine OS cell line K7M2 were sourced from the American Type Culture Collection (ATCC, VA, USA). These cells were cultured in DMEM medium (Gibco, NY, USA) enriched with 10% fetal bovine serum (Gibco, NY, USA) and 1% penicillin-streptomycin (P/S, Gibco, NY, USA) to prevent bacterial contamination. Cultures were maintained at 37 °C in a humidified atmosphere with 5% CO_2_ (23). Cells were used in passages 5 to 10 to ensure biological consistency and experimental reproducibility**.**

### Cell counting kit-8 (CCK8) analysis

Cell viability was assessed using the CCK8 assay. In summary, MG63 and K7M2 cells were plated in 96-well plates at a density of 5×10^3^ cells per well. After adherence, the cells were treated with serially diluted CuB solutions for 24 hr. The CuB stock solution (10 mM) was dissolved in DMSO and diluted in serum-free medium to prepare the working concentrations (0, 0.01, 0.02, 0.04, 0.08, 0.1, 0.2, and 0.4 μM). The final concentration of DMSO in the culture medium was maintained at 0.1% for all experimental conditions. Following treatment, 10 μl of CCK8 solution was added to each well and incubated for one hour. Absorbance at 450 nm was recorded using a microplate reader to quantify cell viability (Thermo Fisher Scientific) (24). Cell viability data were plotted as a dose-response curve with the CuB concentration converted to a logarithmic scale (log[CuB, μM]) on the x-axis. The half-maximal inhibitory concentration (IC_50_) was determined using nonlinear regression analysis in GraphPad Prism 9.0.

### Colony formation assay

MG63 and K7M2 cells (1000 per well) were seeded into 12-well plates. After a 24-hr adherence period, the cells were treated with varying concentrations of CuB (0, 0.05, 0.1, and 0.2 μM) for 24 hr. They were then cultured for one week, with media changes every two days. Following the culture period, the cells were fixed with 4% paraformaldehyde (Beyotime, China) and stained with crystal violet (Beyotime, China) for 15 min to visualize the colonies. A colony was defined as a cluster of at least 50 cells (24, 25). Images of stained colonies were captured, and colony numbers were quantified using ImageJ software.

### Flow cytometry analysis

Apoptosis detection: The detection of apoptotic cells was conducted using the Annexin V-FITC/PI Apoptosis Detection Kit (C1062M, Beyotime, China), following the manufacturer’s protocol. MG63 and K7M2 cells were seeded at a density of 3.5×10^5^ cells per well. Upon reaching approximately 50% confluence, the cells were exposed to varying concentrations of CuB (0, 0.05, 0.1, and 0.2 μM) for 24 hr. After treatment, the cells were rinsed with PBS and stained with Annexin V-FITC and PI (26). Flow cytometry was performed using a flow cytometer, and data were analyzed with FlowJo software (version 10.8). The gating strategy involved excluding debris and doublets based on forward scatter (FSC) and side scatter (SSC) parameters, followed by identifying Annexin V-positive/PI-negative early apoptotic cells and Annexin V-positive/PI-positive late apoptotic cells. Unstained and single-stained controls were used to set appropriate compensation and gating thresholds.

Cell cycle detection: The cell cycle was analyzed using a Cell Cycle and Apoptosis Analysis Kit (C1052, Beyotime, China). After treatment, the MG63 and K7M2 cells were harvested, washed with PBS, and fixed in 70% ethanol at 4 °C for 30 min. They were then incubated with propidium iodide solution at 37 °C in a dark environment for 30 min. The cell cycle distribution was evaluated using a flow cytometer, and data were analyzed with FlowJo software (version 10.8) (27). Doublets and aggregates were excluded based on FSC and SSC parameters, and the PI fluorescence intensity was used to determine the proportion of cells in the G0/G1, S, and G2/M phases. Proper gating controls, including unstained and single-stained samples, were applied to ensure accurate data interpretation.

### Comet assay

The Comet assay was performed following previously described methods(28). MG63 and K7M2 cells were seeded into 12-well plates and treated with CuB (0, 0.05, 0.1, and 0.2 μM) for 24 hr. The Comet assay was conducted to assess DNA double-strand breaks. Following treatment, cells were rinsed with PBS and embedded in low-melting-point agarose. The agarose-cell suspension was then applied dropwise onto pre-coated glass slides and compressed to form a thin layer. Electrophoresis was conducted under conditions of 25 V and 300 mA. Following electrophoresis, the slides were neutralized with Tris-HCl buffer and stained with Hoechst 33342 (#4082) to visualize the nuclei. Fluorescence microscopy was used to capture images to evaluate comet tail formation, and the degree of DNA damage was quantified using ImageJ software.

### Western blot

MG63 and K7M2 cells, along with tissue samples, were lysed using RIPA buffer supplemented with a protease and phosphatase inhibitor cocktail. The lysates were then subjected to surface polyacrylamide gel electrophoresis for protein separation, followed by transfer onto polyvinylidene fluoride membranes. The membranes were subsequently blocked with 5% BSA and incubated with primary antibodies (dilution 1:1000) at 4 °C overnight. Secondary antibodies (dilution 1:5000) were added at room temperature for one hour, followed by visualization of protein bands through enhanced chemiluminescence detection (27). The intensity of the protein bands was quantified using ImageJ software, and the relative expression levels were normalized to the corresponding internal control.

### Real-time fluorescence quantitative polymerase chain reaction (RT-qPCR)

Total RNA was isolated using the RNA-Quick Purification Kit, followed by cDNA synthesis via reverse transcription with the Fast All-in-One RT Kit. Subsequently, qPCR was conducted utilizing the SYBR qPCR Master Mix Kit as per the manufacturer’s protocol, with cycling conditions set to 95 °C (3 min), 60 °C (30 sec), and 95 °C (5 sec), for 40 cycles. The mRNA expression levels were determined using the 2^−^^ΔΔCt^ method (27). The primers used were obtained from Sangon Biotech Co., Ltd, and their sequences are as follows: human (h)-GAPDH forward (5’-3’): GTCTCCTCTGACTTCAACAGCG, h-GAPDH reverse (5’-3’): ACCACCCTGTTGCTGTAGCCAA; h-C-C chemokine ligand 5 (CCL5) forward (5’-3’): CCTGCTGCTTTGCCTACATTGC, h-CCL5 reverse (5’-3’): ACACACTTGGCGGTTCTTTCGG; mouse (m)-GAPDH forward (5’-3’): CATCACTGCCACCCAGAAGACTG, m-GAPDH reverse (5’-3’): ATGCCAGTGAGCTTCCCGTTCAG; m-CCL5 forward (5’-3’): CCTGCTGCTTTGCCTACCTCTC, m-CCL5 reverse (5’-3’): ACACACTTGGCGGTTCCTTCGA.

### Animal experiments

Six-week-old male C57BL/6J mice (18-22 g) were sourced from Nanjing University of Chinese Medicine (Ethics code: ACU240408). The mice were housed in a specific pathogen-free (SPF) environment under controlled conditions (12-hour light/dark cycle, temperature 22±2 °C, humidity 50–60%), and had *ad libitum* access to standard chow and water. For the subcutaneous tumor model, 4×10^6^ K7M2 cells diluted in 100 μl PBS were injected into the right flank of each mouse. Upon reaching a tumor volume of 50 mm^3^, the mice were randomly divided into Control, CuB (0.5 mg/kg), and CuB (1 mg/kg) groups, with six mice per group. Mice in the CuB groups received intraperitoneal injections of CuB daily, and tumor samples were collected on day 15. Tumor volume was measured every 2 days using the formula: length×width^2^/2. Ethical endpoints were defined as tumor volume exceeding 2000 mm³ or significant weight loss (>20%). After the study, mice were euthanized following anesthesia, and samples were collected for follow-up testing.

Six-week-old male C57BL/6J mice (18–22 g) were allocated into four groups (n=6 per group): Control, CuB, anti-PD-L1, and anti-PD-L1+CuB. Tumor transplantation was performed as described above. Mice in the CuB group received daily intraperitoneal injections of CuB (1 mg/kg), those in the anti-PD-L1 group received intraperitoneal injections of anti-PD-L1 (5 mg/kg twice per week), and those in the anti-PD-L1+CuB group received concurrent doses of CuB and anti-PD-L1. Tumor tissues were collected after 15 days.

All animal procedures complied with the guidelines set by the Animal Ethics Committee of Nanjing University of Chinese Medicine.

### Histological analysis

The tumor tissue was preserved in 4% paraformaldehyde and embedded in paraffin before being sectioned into 5 μm slices. Hematoxylin and eosin (H&E) staining was conducted for morphological observation under a microscope (26).

### Immunofluorescent staining

The MG63 and K7M2 cells were immobilized with 4% paraformaldehyde, permeabilized using 0.1% Triton X-100, and then blocked in 5% BSA for one hour. Following this, the cells were exposed overnight at 4 °C to a primary antibody against γ-H2AX (diluted 1:100). Then, the cells were incubated with a secondary antibody (diluted 1:100) for one hour in the dark. Following DAPI staining of the cell nuclei, observations and recordings were made under a laser confocal microscope (26).

### Immunohistochemical staining

After fixation in 4% paraformaldehyde, tumor tissues were sectioned into 5 μm slices, followed by epitope unmasking and inhibition with 5% bovine serum albumin. Incubation with the primary antibody was conducted overnight at 4 °C. After washing with PBS, HRP Polymer was added and incubated at room temperature for 30 min. Subsequently, DAB staining was performed for 15 min, followed by dehydration, clearing, and mounting of the slides for microscopic observation (26).

### Flow cytometry evaluation of tumor-infiltrating immune cells

Tumor tissues were enzymatically dissociated using DNase and Liberase for one hour. The cell suspension was filtered through a 70 μm filter to remove debris and then centrifuged at 1500 rpm. Cell surface markers were stained with mouse-specific FITC anti-mouse CD4 (1:100, BioLegend, 116003), PE/Cyanine7 anti-mouse CD8 (1:100, BioLegend, 140415), PerCP/Cyanine5.5 anti-mouse/human CD11b (BioLegend, 101228), and APC anti-mouse Ly-6G/Ly-6C (Gr-1) (BioLegend, 108411). For granzyme B (Grzm) and IFN-γ staining, cells were fixed with brefeldin A solution (BioLegend, 420601), monensin solution (BioLegend, 420701) fixation/permeabilization kit and (BD Bioscience, 554714) for 30 min, then stained with Alexa Fluor® 647 anti-human/mouse granzyme B (BioLegend, 515406) and Alexa Fluor® 488 anti-mouse type I interferons (IFN-γ) (BioLegend, 505813). Corresponding isotype controls were also included. All antibodies were diluted at a 1:100 ratio in PBS. Flow cytometric analysis was then performed using the Attune NxT Flow Cytometer (Thermo Fisher Scientific). Data were processed and analyzed using FlowJo software. The gating strategy was employed for flow cytometric analysis to identify the target cell populations. First, FSC and SSC were used to exclude debris and dead cells by applying a gate on the FSC vs SSC plot. Then, single cells were selected by gating on the FSC-A vs FSC-H plot to exclude doublets. Afterward, live cells were selected by excluding dead cells using a viability marker. To identify the immune cell populations, gating was performed based on the specific surface markers: CD4+ T cells were gated on FITC anti-mouse CD4, CD8+ T cells were gated on PE/Cyanine7 anti-mouse CD8, myeloid cells were gated on CD11b, and neutrophils were gated on Ly-6G/Ly-6C (Gr-1). For intracellular staining of granzyme B and IFN-γ, cells were further gated based on the expression of granzyme B (Alexa Fluor® 647) and IFN-γ (Alexa Fluor® 488). Isotype controls were used to define the background staining, ensuring specificity in the analysis.

### Statistical analysis

Statistical analyses were performed using GraphPad Prism 9.0. Data are presented as mean ± standard error of the mean (SEM). One-way ANOVA was utilized to compare multiple groups. Statistical significance was defined at *P*<0.05. Results were obtained from at least three independent experiments, each test performed in triplicate.

## Results

### CuB suppresses OS cell viability, induces apoptosis, and promotes cell cycle arrest

Initially, we examined the effects of CuB on OS cells. The CCK8 assay confirmed a potent inhibitory effect, with IC_50_ values of 0.1038 μM for MG63 cells and 0.1154 μM for K7M2 cells (Figure 1B). Subsequently, we selected concentrations corresponding to 1/2 IC_50_, IC_50_, and 2×IC_50_ for further experiments. Colony formation assays further demonstrated that CuB inhibited the clonogenic potential of MG63 and K7M2 cells in a dose-dependent manner (Figure 1C). Additionally, flow cytometry analysis revealed that CuB induced apoptosis and cell cycle arrest at the G2/M phase in both MG63 and K7M2 cells (Figure 1D, E). Collectively, these findings indicate that CuB suppresses OS cell survival, induces apoptosis, and promotes cell cycle arrest.

### CuB initiates a DNA damage response in OS cells

**Figure 2:**
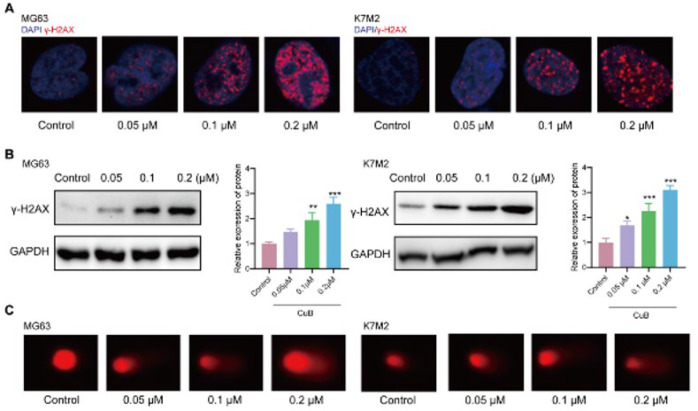
CuB initiates a DNA damage response in OS cells

In cancer therapy, inducing DNA damage in tumor cells to promote apoptosis or inhibit proliferation is a critical strategy (29). To determine whether CuB induces DNA damage, we evaluated the expression of γ-H2AX, an early marker of DNA damage, in OS cells following CuB treatment. Immunofluorescent staining and Western blot analysis demonstrated a marked increase in γ-H2AX levels in MG63 and K7M2 cells upon CuB exposure (Figure 2A, B). To further evaluate DNA damage, we conducted a comet assay, with the comet tail moment serving as an indicator of DNA fragmentation. Compared to untreated controls, CuB-treated cells exhibited a significant increase in comet tail moments (Figure 2C), confirming enhanced DNA damage. Collectively, these findings provide compelling evidence that CuB induces DNA damage in OS cells.

### CuB activates the STING signaling pathway in OS cells

**Figure 3:**
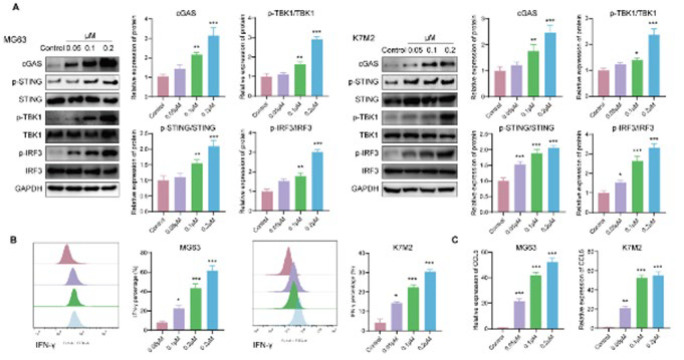
CuB activates the STING signaling pathway in OS cells

The cGAS/STING pathway, activated by cytosolic DNA, has been identified as a crucial initiator of antitumor immune responses (30). We postulated that CuB could stimulate STING-dependent signaling by inducing DNA damage. To investigate this, we examined the impact of CuB on STING signaling in MG63 and K7M2 cells. Western blot analysis revealed significant increases in cGAS, p-STING, p-TBK1, and p-IRF3 levels in CuB-treated MG63 and K7M2 cells compared to the control group (Figure 3A). Emerging evidence suggests that STING activation leads to increased production of IFN-γ. Consistently, CuB treatment markedly elevated IFN-γ levels in MG63 and K7M2 cells (Figure 3B). Furthermore, CCL5, a chemokine regulated by IRF3, was significantly up-regulated at the mRNA level following CuB treatment, as demonstrated by RT-qPCR (Figure 3C). 

These results suggested that CuB may exert anti-OS effects by activating the STING signaling pathway through DNA damage.

### CuB suppresses tumor growth in a murine OS xenograft model

**Figure 4:**
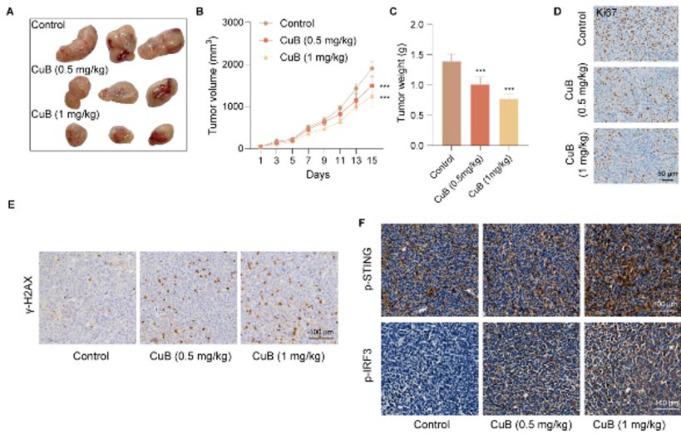
CuB suppresses tumor growth in a murine OS xenograft model

We established a mouse OS xenograft model to further evaluate CuB’s antitumor effects. Compared to the control group, CuB-treated mice (0.5 or 1 mg/kg) demonstrated a significant reduction in tumor volume and weight (Figure 4A-C). Immunohistochemical staining of Ki67 in tumor tissues revealed a marked decrease in Ki67-positive cells after CuB treatment (Figure 4D).

We conducted immunohistochemical staining on tumor tissues to explore whether CuB-induced tumor suppression is linked to DNA damage and the activation of the STING signaling pathway. The results demonstrated a significant increase in the expression levels of γ-H2AX, p-STING, and p-IRF3 in the tumor tissues of mice treated with CuB (Figures 4E and F).

These results suggest that CuB inhibits OS tumor progression *in vivo*, potentially by inducing DNA damage and activating the STING pathway.

### CuB enhances immune cell infiltration in mouse tumor tissue

**Figure 5:**
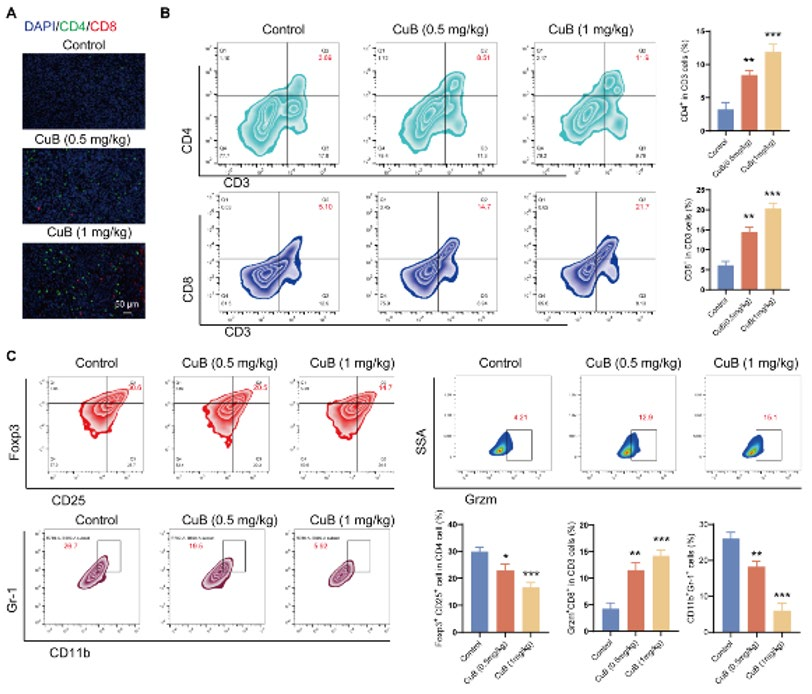
CuB enhances immune cell infiltration in mouse tumor tissue

Recent evidence suggests that DNA resulting from DNA damage-induced production of intracellular cytosolic DNA can trigger innate immune responses. We assessed immune cell infiltration in mouse tumor tissues to further validate the impact of the CuB-activated STING signaling pathway on immune responses in the tumor microenvironment. Immunofluorescence staining revealed a significant increase in both CD8+ T cells and CD4+ T cells in the tumor tissues of mice treated with CuB (0.5 or 1 mg/kg) (Figure 5A). The level of Grzm in tumor-infiltrating lymphocytes (TILs) indicates cytotoxic T-cell activity. CuB treatment enhanced Grzm production by CD8+ T cells. Flow cytometry analysis showed a notable increase in the proportion of helper T cells in the tumor tissues of CuB-treated mice compared to the control group (Figure 5B). Additionally, CuB treatment reduced the proportion of regulatory T cells (Tregs) in tumor tissues (Figure 5C). Myeloid-derived suppressor cells (MDSCs) are known to suppress the function of immune cells, such as T lymphocytes, NK cells, and antigen-presenting cells, promoting immune tolerance and tumor immune escape, therefore fostering an immunosuppressive microenvironment. Flow cytometry analysis showed that CuB increased the proportion of cytotoxic T lymphocytes (CTLs) while reducing MDSCs in the tumor tissues (Figure 5C). These results suggest that CuB-mediated activation of the STING signaling pathway exerts antitumor effects by enhancing immune cell infiltration into the tumor microenvironment.

### CuB enhances the anticancer effect of anti-PD-L1 in mouse OS

**Figure 6:**
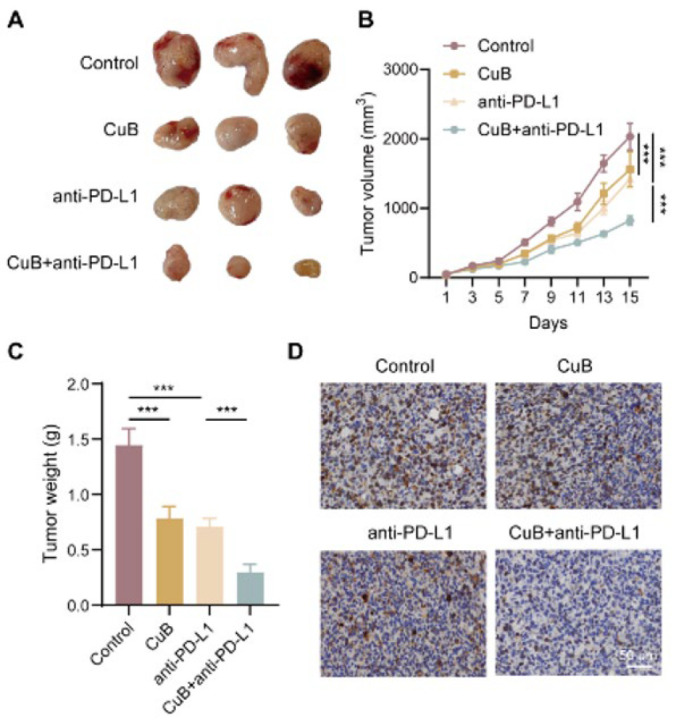
CuB enhances the anticancer effect of anti-PD-L1 in mouse OS

Combination therapy with anti-PD-L1 and other agents has demonstrated higher response rates than PD-L1 monotherapy, significantly enhancing both response and survival rates in cancer patients. To assess the synergistic effects of CuB in combination with anti-PD-L1 on mouse xenograft tumors, we treated mice with CuB, anti-PD-L1, or a combination of both. Compared to the control group, mice receiving either CuB or anti-PD-L1 therapy exhibited a notable decrease in tumor size and weight. Notably, the combination therapy resulted in an even greater tumor volume and weight reduction than either treatment alone (Figure 6A-C). Additionally, immunohistochemical analysis revealed a significant decrease in Ki67-positive cells within the tumors of mice undergoing combination therapy (Figure 6D). 

These results indicate that CuB enhances the anticancer efficacy of anti-PD-L1, leading to more effective inhibition of OS tumor growth.

## Discussion

OS is highly aggressive, with a strong propensity for metastasis and local recurrence, necessitating multimodal treatment strategies. Despite advancements in chemotherapy and targeted therapies, resistance and immune evasion remain significant challenges (3). This study demonstrates that CuB exerts anti-OS effects by inducing DNA damage, activating the STING signaling pathway, and enhancing antitumor immune responses.

Our findings reveal that CuB significantly induces DNA damage in OS cells, as evidenced by increased γ-H2AX expression and comet assay results. DNA damage is a well-established trigger of tumor cell apoptosis and cell cycle arrest; however, its role in modulating tumor immunity has recently gained attention (31, 32). One key pathway linking DNA damage to immune activation is the cGAS-STING axis, which detects cytosolic DNA and initiates type I interferon responses (19, 33). Here, we provide evidence that CuB activates the STING pathway in OS cells, as demonstrated by increased expression of key signaling proteins (p-STING, p-TBK1, and p-IRF3). This activation is accompanied by elevated production of IFN-γ and CCL5, two critical mediators of antitumor immunity (34). These findings suggest that CuB induces direct cytotoxic effects on OS cells and contributes to the activation of immune signaling pathways, potentially influencing the tumor immune microenvironment.

The *in vivo* results further support this notion. CuB treatment significantly suppressed tumor growth in OS-bearing mice, and immunohistochemical analysis confirmed the upregulation of γ-H2AX and activation of the STING pathway in tumor tissues. Notably, CuB treatment also promoted immune cell infiltration within the tumor microenvironment. Flow cytometry analysis demonstrated an increased presence of CD4+ T cells, CD8+ T cells, helper T cells, and CTLs, while Tregs and MDSCs were decreased. These findings indicate that CuB may help to shape a tumor microenvironment that favors immune activation, further supporting its potential role in OS therapy.

An intriguing aspect of our study is the observed interplay between CuB-induced STING activation and PD-L1/PD-1 signaling. Previous studies suggest that STING activation can enhance tumor immunogenicity and improve responses to immune checkpoint blockade(35). In line with this, our combination therapy experiments demonstrated that CuB enhances the efficacy of anti-PD-L1 treatment in OS-bearing mice, leading to superior tumor suppression compared to monotherapy. This suggests a potential synergistic effect between CuB and immune checkpoint inhibitors.

Despite these promising results, several questions remain. The tumor microenvironment is highly complex, and additional studies are needed to explore how CuB affects other immune cell populations, including macrophages and dendritic cells. While our preclinical data strongly support CuB’s potential as an OS therapy, clinical validation is essential. Future studies should investigate CuB’s pharmacokinetics, toxicity profile, and possible combinatorial strategies in clinical settings.

(A) Chemical structure of CuB. (B) CCK8 assay assessing the viability of MG63 and K7M2 cells following CuB treatment (n=4). (C) Colony formation assay evaluating CuB's inhibitory effect on the clonogenic potential of MG63 and K7M2 cells (n=4). (D) Flow cytometry analysis of CuB-induced apoptosis in MG63 and K7M2 cells, with apoptosis quantified as the sum of Q2 and Q3 fractions (n=4). (E) Flow cytometry analysis of CuB-induced changes in cell cycle distribution in MG63 and K7M2 cells (n=4). Data are presented as the mean ± SD. *P<0.05, **P<0.01, *** *P*<0.001.

(A) Immunofluorescence staining of γ-H2AX in MG63 and K7M2 cells following CuB treatment (scale bar = 20 μm). (B) Representative Western blot analysis of γ-H2AX protein expression in CuB-treated MG63 and K7M2 cells (n=3). (C) Representative comet assay images following CuB treatment. Data are presented as the mean ± SD. *P<0.05, *** *P*<0.001.

(A) Representative Western blot analysis showing γ-H2AX protein expression following CuB treatment in MG63 and K7M2 cells (n=3). (B) Flow cytometry analysis of IFN-γ levels (n=4). (C) Relative mRNA expression of CCL5, as determined by RT-qPCR (n=4). Data are presented as the mean ± SD. *P<0.05, **P<0.01, *** *P*<0.001.

(A) Representative images of xenograft tumors from each group. (B) Tumor volume. (C) Weight measurements for each group (n=6). (D) Immunohistochemical staining of Ki67 in tumor tissues (scale bar = 50 μm). (E) Representative images of γ-H2AX immunohistochemical staining in tumor tissues from xenograft mice (scale bar = 100 μm). (F) Representative images of p-STING and p-IRF3 immunohistochemical staining in tumor tissues from xenograft mice (scale bar = 100 μm). Data are presented as the mean ± SD. *** *P*<0.001.

(A) Representative images of CD4 and CD8 immunofluorescence staining of mouse tumor tissue (scale bar = 50 μm). (B) Flow cytometry analysis of the proportions of helper T cells and CTLs in mouse tumor tissues (n=6). (C) Flow cytometry analysis of the proportions of Tregs, Grzm-positive CTLs, and MDSCs in mouse tumor tissues (n=6). Data are presented as the mean ± SD. *P<0.05, **P<0.01, *** *P*<0.001.

(A) Representative images of xenograft tumors from each group. (B) Tumor volume. (C) Weight statistics for each group (n=6). (D) Immunohistochemical staining of Ki67 in tumor tissues (scale bar = 50 μm). Data are presented as the mean ± SD. *** *P*<0.001.

## Conclusion

In summary, both *in vitro* and *in vivo* results demonstrate that CuB exerts anti-OS effects by inducing DNA damage and activating the STING signaling pathway. By activating the STING signaling pathway, CuB promotes the infiltration of immune cells in mouse tumor tissue and synergizes with anti-PD-L1 to produce anti-OS effects. These findings underscore the potential of CuB as an adjuvant therapy for OS.

## Data Availability

All data generated or analyzed during this study are included in this published article.

## Declaration

The authors have not used AI tools or technologies to prepare this manuscript.
